# Mutations in *UCP2* in Congenital Hyperinsulinism Reveal a Role for Regulation of Insulin Secretion

**DOI:** 10.1371/journal.pone.0003850

**Published:** 2008-12-09

**Authors:** M. Mar González-Barroso, Irina Giurgea, Fredéric Bouillaud, Andrea Anedda, Christine Bellanné-Chantelot, Laurence Hubert, Yves de Keyzer, Pascale de Lonlay, Daniel Ricquier

**Affiliations:** 1 Université Paris Descartes Site Necker-enfants Malades, CNRS UPR 9078 BIOTRAM, Paris, France; 2 Université Paris Descartes Site Necker-enfants Malades, INSERM U781 and Departments of Pediatrics, Hôpital Necker-Enfants Malades Assistance Publique Hôpitaux de Paris, Paris, France; 3 Centro de Investigaciones Biológicas, CSIC, Madrid, Spain; 4 Département de Génétique, Groupe Hospitalier Pitié-Salpétrière Assistance Publique Hôpitaux de Paris, Paris, France; Stanford University School of Medicine, United States of America

## Abstract

Although the most common mechanism underlying congenital hyperinsulinism is dysfunction of the pancreatic ATP-sensitive potassium channel, the pathogenesis and genetic origins of this disease remains largely unexplained in more than half of all patients. UCP2 knockout mice exhibit an hyperinsulinemic hypoglycemia, suggesting an involment of UCP2 in insulin secretion. However, a possible pathogenic role for UCP2 protein in the development of human congenital hyperinsulinism or of any human disease has not yet been investigated. We studied ten children exhibiting congenital hyperinsulinism, without detectable mutations in the known congenital hyperinsulinism-causing genes. Parental-inherited heterozygous UCP2 variants encoding amino-acid changes were found in two unrelated children with congenital hyperinsulinism. Functional assays in yeast and in insulin-secreting cells revealed an impaired activity of UCP2 mutants. Therefore, we report the finding of UCP2 coding variants in human congenital hyperinsulinism, which reveals a role for this gene in the regulation of insulin secretion and glucose metabolism in humans. Our results show for the first time a direct association between UCP2 amino acid alteration and human disease and highlight a role for mitochondria in hormone secretion.

## Introduction

Congenital hyperinsulinism (CHI, OMIM 256450) is a rare genetic disorder characterised by hyperinsulinemic hypoglycemia caused by unpredictable excessive insulin secretion. The severity of the disease varies from a mild form to a severe form which may require surgical removal of the pancreas to protect the brain from damage due to recurrent hypoglycemia. Hyperinsulinism may be caused by a range of biochemical disturbances and molecular defects. Most severe CHI are due to mutations in both genes *ABCC8* and *KCNJ11* encoding ATP-dependent potassium channel subunits SUR1 and Kir6.2 [1]–[3]. Other mutations have also been identified in several other genes [4]–[9]. However, in approximately 40–50% of all CHI cases, no mutation has been found in any of these known genes, suggesting the existence of other disease-associated genes, especially among those sensitive to diazoxide treatment [10], [11].

The mitochondrial uncoupling protein 2 (UCP2), a member of the UCP family, is widely expressed in tissues, including pancreatic islets [12]. Due to the high similarity to UCP1, it has been proposed that active UCP2 induces regulated leak of protons across the inner mitochondrial membrane, and as a result, uncoupled mitochondrial oxidative metabolism from ATP synthesis [13]–[15]. Consequently, although it is debated, UCP2 is predicted to reduce the ATP yield from substrate oxidation. Since in pancreatic β-cells, ATP is a key signal for glucose sensing and insulin secretion, it was predicted that UCP2 could negatively regulate glucose-stimulated insulin secretion (GSIS). Overexpression of UCP2 in isolated rat pancreatic cells decreased ATP content and inhibited GSIS [16], [17]. Similarly, *Ucp2* knockdown by RNAi in pancreatic beta cells enhanced GSIS [18]. Data coming from rodents models and animal experiments support the idea that changes in UCP2 expression and/or activity could contribute to beta cell dysfunction and to the pathogenesis of diabetes [19], [20]. Whole-body partial inhibition of UCP2 expression by antisense oligonucleotide treatment reversed hyperglycemia in models of diabetes and insulin resistance [21]. Consistent with this, inhibition of UCP2 activity by genipin, a natural compound from *Gardenia*, reversed high glucose- and obesity- induced beta cell function [22]. Also supporting a role for UCP2 in the regulation of insulin secretion have been studies performed in *Ucp2* knockout mice. These mice have been shown to exhibit hypoglycaemia as a result of increased GSIS associated with higher ATP levels in isolated islets [23]. When *Ucp2* knockout mice were crossed with *ob/ob* mice, the offspring displayed a significant improvement in glucose tolerance [23]. In addition, there is also evidence in humans that a functional polymorphism in the *Ucp2* gene promoter correlated with lower insulin secretion and a higher risk for the development of type 2 diabetes [24]–[26]. Given that many patients with CHI do not have mutations in known CHI-associated genes, and given the implicated role of UCP2 in the regulation of insulin secretion we investigated UCP2 as a new candidate gene involved in human hyperinsulinic states.

## Materials and Methods

### Patients and genotype analyses

Written informed consent was obtained from all human participants before collecting blood for DNA extraction. The study was approved by Necker-Enfants Malades Hospital review board. Ten CHI children with no detectable mutations by DNA direct sequencing in the *ABCC8*, *KCNJ11*, *GCK* or *GLUD1* genes were included in this study. These ten children presenting repeated hypoglycemias (ranging from 0.9 to 2.3, reference range >2.7 mmol/l) associated to high insulin levels (ranging from 5.5 to 40 mU/l, reference range <0.4 in hypoglycemia), consistent with the diagnosis of congenital hyperinsulinism. Hypoglycaemia occurred either at birth (5/10 cases) or in infancy (5/10 cases). Those who underwent surgery (4/10) were subsequently classified as either diffuse CHI (n = 1) according to previously described histological criteria [27] or atypical CHI (n = 3), a mosaic of heterogeneous hyperfunctional islets. The other patients (6/10) were treated medically with diazoxide. The medical sensitivity to diazoxide was defined as the complete normalisation of blood glucose (>3 mmol/l), measured before and after each meal, in patients fed normally. After that intravenous glucose and other medications had been stopped for at least five consecutive days. Resistance to medical treatment was defined as two confirmed blood glucose measurements lower than 3 mmol/l in a 24-hours period.

Transhepatic selective pancreatic venous catheterization [28] was performed to detect diffuse insulin oversecretion in the whole pancreas or to localize the insulin hypersecretion to a discrete part of the pancreas. Diazoxide and all other drugs were stopped 7 days before catheterization. In patients with focal CHI, transhepatic selective pancreatic venous catheterization typically gave rise to localised and markedly elevated insulin and C-peptide levels in the territory of the lesions, with low levels in the remaining pancreas. Diffuse CHI was considered when increased insulin and C-peptide levels were found throughout the whole pancreas.

The human *UCP2* gene was screened by denaturating high performance liquid chromatography (DHPLC). Genomic DNA was purified from peripheral blood leukocytes according to standard procedures. Primers designed for amplifying *UCP2* exons and exon-intron boundaries are indicated in Table 1. PCR amplifications consisted of an initial denaturation at 94°C for 5 min, followed by 30 cycles at 94°C for 30 sec, 65°C for 30 sec and 72°C for 1 min, and a final extension at 72°C for 10 min. The PCR products were denatured at 95°C for 5 min and allowed to cool to 25°C for the formation of heteroduplexes. DHPLC was carried out using a Transgenomic WAVE DHPLC and DNASep column (Transgenomic™) as previously described [29] . For analysis, a flow rate of 0.9 ml/min was used, with the buffer (0.1 M triethylammonium acetate, 25% acetonitrile) gradient increased for 3.5 min, as indicated in table 1. PCR products displaying abnormal DHPLC patterns were purified and directly sequenced using the PRISM™ Ready Reaction Sequencing Kit (Perkin-Elmer) on an automatic sequencer (ABI 3100). All procedures were approved by the review board of the Necker-Enfants Malades Hospital.

**Table 1:** PCR an DHPLC conditions

Primer	Sequence of the primer	Melting temperature (°C)	Gradient of acetonitrile (% tampon B)	Temperature of the mobile phase (°C)
Pr-exon 1 S Pr-exon 1 AS	5′ agaggaagtgcacttaagac 3′ 3′ gagtacaaacttgggtggga 5′	58	*Not studied in DHPLC*	
exon 2 S exon 2 AS	5′ gcatggattgggtgggcttg 3′ 3′ ctggaaagagccacttcactg 5′	60	*Not studied in DHPLC*	
exon 3 S exon 3 AS	5′ ttagggaaggtgagtttggg 3′ 3′ tctcgatgctccaaacactg 5′	60	52–60 49–57	60 62
exon 4 S exon 4 AS	5′ caaagacacagacccctcaa 3′ 3′ gatcgtggggcctaaaaaac 5′	60	56–64 54–62 52–60	59 61 65
exon 5 S exon 5 AS	5′ ggtagaaaatgagtgcaagcc 3′ 3′ tgtaggaggaggaagatcct 5′	60	56–64 53–61	61 64
exon 6 S exon 6 AS	5′ gcctctggaaaggtgtgtac 3′ 3′ cttactctccctgcaaaggg 5′	62	57–65 49–57	60 62
exon 7 S exon 7 AS	5′ ctggaatgatgggtgaagact 3′ 3′ gctactcacttccaggtggt 5′	60	57–65 54–62	60 62
exon 8 S exon 8 AS	5′ ggttggattgaataccaggc 3′ 3′ agaaaaggaaagcatggccc 5′	60	57–65 49–57	61 62

### Vector for yeast expression and site-directed mutagenesis

Wild-type and mutated human UCP2 (hUCP2) cDNA sequences (as found in patient 1 and patient 2 ) were introduced into the pYeDP-1/8–10 vector as described previously [30] . Site-directed mutagenesis was performed using the QuickChange Site-Directed Mutagenesis Kit according to the manufacturer's instructions (Stratagene, La Jolla, California, USA). Sequences of the synthetic oligonucleotides used were as follows (5′ to 3′) : GAAGGGTTCCGaGatCTCTGGAAAGGG(G174D) ; GAAGGGTTCCGcGaGCgTCTGGAAAGGG(L175V) ; TTGCTCGTAATGatATcGTCAACTGTGCTGA(A187D) ; GAAGGAGGGGCCtCGAGgaTTCTACAAAGGGT(A268G). Plasmids were sequenced to confirm the introduction of expected mutations by automatic DNA sequencing.

Wild type and mutated human UCP2 cDNA sequences cloned into the pYeDP-1/8–10 vector were introduced in the diploid *Saccharomyces cerevisiae* strain W303 by electroporation. Expression levels and mitochondrial targeting were analyzed by western blot of yeast mitochondrial preparations with polyclonal hUCP2 605 antibody against human UCP2 [31].

### Isolation of yeast mitochondria and spheroplasts

Mitochondria were isolated from transformed yeasts grown overnight in SG medium (2% galactose, 0.67% yeast nitrogen base, 0.1% casamino acids, 20 mg/L tryptophan and 40 mg/L adenine) as previously described [30] . Yeast spheroplasts were also prepared from overnight cultures growing in SG medium according to Averet et al. [32] .

### Proton leak measurements in permeabilized yeast spheroplasts

Spheroplasts were permeabilized with 0.2 mg/ml nystatin at 30°C in respiration medium containing 1 M sorbitol, 0.5 mM EGTA, 2 mM MgSO_4_, 1.7 mM NaCl, 10 mM potassium phosphate (pH 6.8), 0.1% BSA. Mitochondrial respiration was measured at 20°C in the presence of 6 mM NADH with an oxygen electrode. Mitochondrial membrane potential was simultaneously determined with a home-made tetraphenylphosphonium (TPP^+^) electrode prepared according to published procedures [33] . The amount of spheroplasts used in the experiments was adjusted every day to obtain always the same maximal respiratory rate in the presence of an uncoupler (1 µM FCCP). The initial state 4 of respiration was obtained in the presence of 10 µg/ml oligomycin . Respiratory chain activity was gradually inhibited by the addition of increasing concentrations of potassium cyanide (ranging from 1 to 300 µM).

### Adenovirus generation for expression of UCP2 mutants in insulin-secreting mammalian cells

Recombinant adenoviruses were generated according to the homologous recombination pAdEasy system as described [34] . The full-length hUCP2 cDNA as well as the cDNAs carrying UCP2 mutations equivalent to patient 1 and patient 2 and the rat UCP1 cDNA were cloned into the shuttle vector pAdTrack-CMV. This vector allows the production of green fluorescent protein (GFP)-trackable viruses, as it contains the *GFP* gene in tandem with the transgene. Expression of both genes is under control of different cytomegalovirus promoters. Homologous recombination between the pAdEasy-1 and pAdTrack-CMV vectors and production of the wild type (AdhUCP2), mutated hUCP2 (AdhU2Patient1 and AdhU2Patient2) and rUCP1 (AdrUCP1) adenoviruses were performed at the Laboratoire de Thérapie Génique (INSERM, Nantes-France). An adenovirus containing the *GFP* gene and the inverted sequence of human UCP2 cDNAwas used as control (Adcontrol).

### Cell culture and adenoviral infection

The rat insulinoma cell line INS-1E was provided by Dr. P. Maechler (University of Geneva, Switzerland) [35]. Cells were seeded in 6-well plates and maintained in RPMI 1640 medium containing 10% FCS, 10 mM HEPES, 1 mM sodium pyruvate, 50 µM 2-mercaptoethanol, 4 mM glutamine, 100 IU/ml penicillin and 100 µg/ml streptomycin at 37°C under 5% CO_2_. The cells were infected at near confluence with Adcontrol, AdhUCP2 and AdrUCP1 viral stocks at 5.2×10^6^ plaque-forming units (pfu)/ml. To obtain similar amounts of heterologous protein expressed, the cells were infected with 1.3×10^6^ pfu/ml of AdhUCP2Patient1 or 2.6×10^6^ pfu/ml of AdhUCP2Patient2 viral stocks. The virus was applied to cells for 4 h in the absence of serum. The cells were then washed of virus and supplemented with 10% serum-containing RPMI 1640 for 48 h.

### Mitochondrial isolation from INS-1E cells and western blot analysis

Cells were washed twice with PBS, scraped and collected by centrifugation at 2000 *g* for 5 min at 4°C. Mitochondria were isolated from cells. 10 µg of mitochondrial proteins were resolved by SDS-PAGE and transferred to nitrocellulose membranes before inmunoblotting. UCP2 protein was detected with the anti-hUCP2 (605 batch) [31] or anti-UCP2 (C-20) (Santa Cruz Biotechnology, Inc, Santa Cruz, CA, USA) antibodies. For UCP1 detection a polyclonal anti-rat UCP1 antibody prepared in the laboratory was used. Anti-VDAC/porin antibody (Sigma-Aldrich, St Louis, MO, USA) was used as loading control of equivalent amount of mitochondrial proteins.

### Assay of insulin secretion

24 h after adenoviral infection, INS-1E cells were cultured overnight in RPMI 1640 medium containing 5 mM glucose and 10% FCS. Cells were washed and preincubated for 1 h in RPMI medium without glucose and 1% FCS. The medium was replaced by 1 ml of Krebs-Ringer bicarbonate buffer (KRBH) (135 mM NaCl, 3.6 mM KCl, 5 mM NaHCO_3_, 0.5 mM NaH_2_PO_4_, 0.5 mM MgCl_2_, 1.5 mM CaCl_2_, 10 mM HEPES pH 7.4 and 0.1 % fatty acid-free BSA) with 2.5 mM glucose. When indicated, 32 µM retinoic acid was added to KRBH medium. After 30 min incubation at 37°C, the cells were incubated for another 30 min with KRBH containing 15 mM glucose. When indicated, 32 µM retinoic acid was added to KRBH medium. The medium was collected and cleared of non adherent cells. Insulin content in the supernatants was determined by radioimmunoassay for rat insulin (Linco Research, St. Charles, MO). Total cell protein was determined for each well with the *DC* protein assay (Bio-Rad).

### Statistical Analysis

Data are presented as means±S.E.M. Results were compared by Student's paired *t* test or one-way ANOVA, where applicable. The level of significance were indicated as * *p*<0.01 and ** p<0.001.

## Results

### Molecular studies

**Figure 1:**
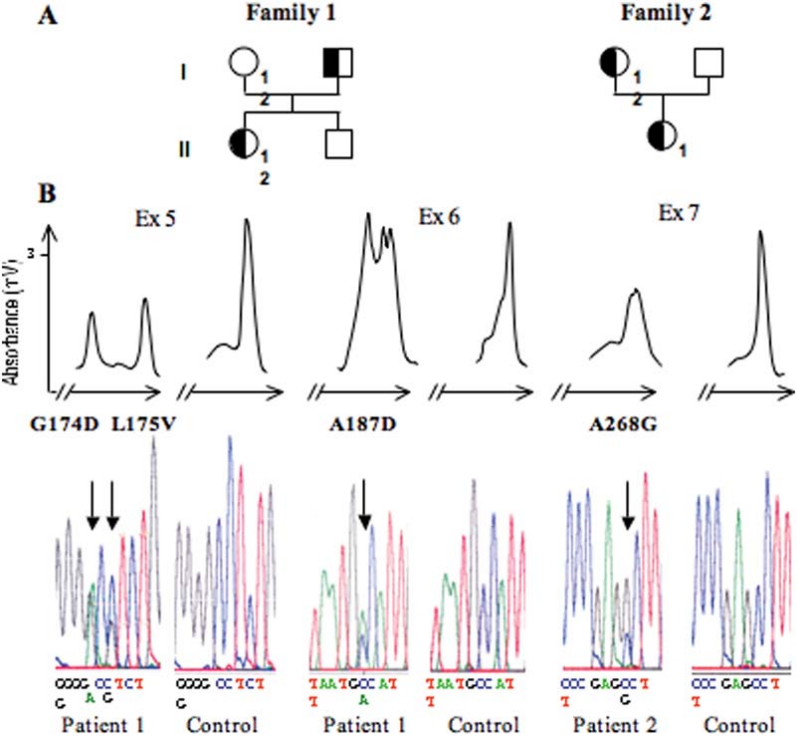
Pedigree of Families 1 and 2 with missense heterozygous sequence variations in the UCP2 gene. A. Pedigree: Family 1 (F1): Patient 1 had neonatal hypoglycaemia, was sensitive to diazoxide and resolved spontaneously at age 1 year. Her parents did not present clinical signs of hypoglycaemia. Family 2 (F2): Patient 2 developed hypoglycaemia at 8 month of age, sensitive to diazoxide. His mother presented seizures in infancy and occasional hypoglycaemia. Individuals carrying heterozygous *UCP2* sequence variations are indicated by partly closed and partly open symbols. B. DHPLC patterns (upper part) and corresponding DNA sequences (lower part) of family 1 and family 2.

Details of hypoglycemias and high insulin levels in CHI patients are given in Materials and Methods section. PCR fragments of UCP2 exons were synthetized from peripheral blood leukocyte of 10 children with no detectable mutations by DNA direct sequencing in the *ABCC8*, *KCNJ11*, *GCK* or *GLUD1* genes. Each PCR reaction was subjected to DHPLC analysis and their corresponding elution profiles were analyzed. Abnormal DHPLC pattern was detected for 2 unrelated patients, born to non-consanguineous parents (Figure 1). DNA sequence analysis identified respectively 3 and 1 heterozygous single nucleotide changes in patients 1 and 2, all of them leading to an amino acid change. Mutations of patient 1 consisted of a G to A transition at nt 521 changing a conserved glycine into an aspartic acid (G174D), a C to G transversion at 523 nt changing a conserved leucine into a valine (L175V) and a C to A transition at nt 560 changing a conserved alanine into an aspartic acid (A187D, Figure 1). All mutations were inherited from her father. Patient 2 mutation, inherited from her mother, was a C to G transversion at nt 803 changing a conserved alanine into a glycine (A268G, Figure 1).

**Figure 2:**
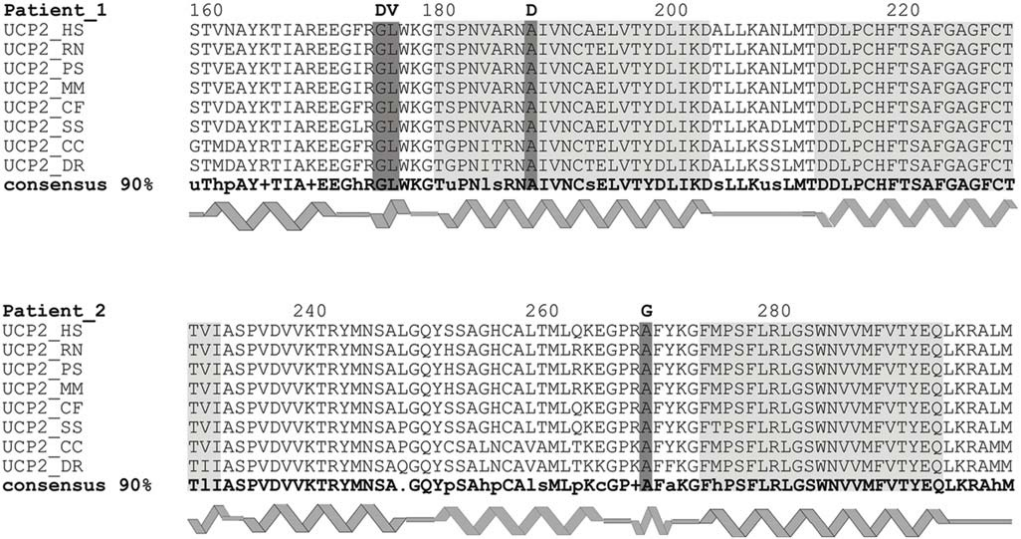
Amino acid changes in UCP2 of patients 1 and 2 and conservation among species. Comparison of the aminoacid sequences of the second moiety of UCP2 from *Homo sapiens* (GenBank Accession NP003346), *Rattus norvegicus* (GenBank Accession NP062227), *Phodopus sungorus*(GenBank Accession AAG33984), *Mus musculus* (GenBank Accession NP035801), *Canis familiaris* (GenBank Accession BAA90457), *Sus scrofa* (GenBank Accession NP999454), *Cyprinus carpio* (GenBank Accession CAB46248), *Danio rerio* (GenBank Accession NP571251). The multiple sequence alignment was carried out with Clustal X (v. 1.8) analysis software. The UCP2 consensus sequence was calculated from the site http://coot.embl.de/cgi/consensus. The ribbon cartoon symbolizes the predicted α-helix domains according to the 3D structure of the ADP/ATP carrier [51]. The putative transmembrane motifs are marked in grey background and the residues mutated in the two patients in dark grey background. Bold letters in the upper line represent the aminoacids found in Patient 1 and Patient 2.

The amino acid changes identified in these two patients were not found in 100 control subjects. All these residues are highly conserved in UCP2 protein from different species (Figure 2). The amino acids G174 and L175, mutated in patient 1, are localised at the end of the second matrix loop, just before nucleation of the fourth transmembrane α-helix. The residue A187, involved also in the first patient, is into the fourth transmembrane α-helix. The A268, mutated in the patient 2, is located at the end of the third matrix hydrophilic loop, before the sixth transmembrane domain of UCP2 (Figure 2).

Patient 1, a girl, was born at gestational age of 38 weeks (birth weight 3590 g, length 51.5 cm, head circumference 34 cm) from two unrelated Caucasian parents. She developed neonatal hypoglycaemia and was successfully treated by diazoxide during 1 year (data not shown). After this time, diazoxide was stopped without a recurrence of hypoglycaemia. The parents were healthy.

Patient 2, also a girl, was born at gestational age of 37 weeks (birth weight 2850 g, birth height 48 cm, head circumference 34 cm) from two unrelated Caucasian parents. She presented with seizures at 8 months of age which revealed hypoglycaemia. Clinical and biological investigations confirmed the diagnosis of hyperinsulinism. Pancreatic venous sampling revealed a diffuse insulin secretion suggesting a diffuse form of CHI that was successfully treated by diazoxide for a period of 2 years. The child was not seen in the department after these two years. Her mother was found to be occasionally hypoglycaemic at adult age and has been treated by an antiepileptic drug for seizures during infancy.

### Functional studies in yeast

**Figure 3:**
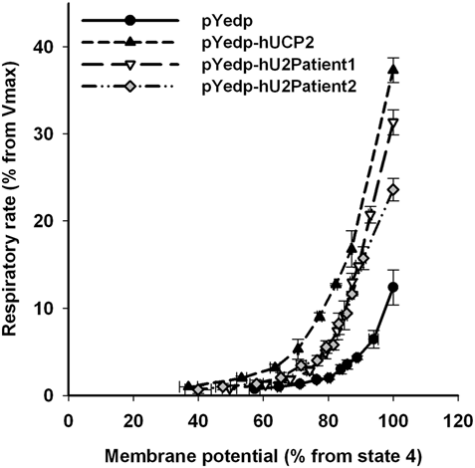
Comparison of the proton leak kinetics of yeast spheroplasts. Spheroplasts were isolated from control (pYeDP plasmid, black circles), wild type UCP2 (pYeDP-hUCP2, black triangles) and mutated (as found in patient 1 and 2) UCP2 (pYeDP-hU2Patient1, open diamonds and pYeDP-hU2Patient2, grey squares) yeasts as described in “Materials and Methods” section. Mitochondrial membrane potential and respiratory rate were simultaneously recorded and varied by titration with potassium cyanide in the presence of NADH. These experiments were performed in the presence of oligomycin using the same amount of respiratory chain for the different types of spheroplasts as determined from the maximal respiratory rate in the presence of FCCP (for details see “Materials and Methods” section). Respiratory rate (*y* axis) is represented as the percentage from the maximal respiratory rate (Vmax) in the presence of FCCP. Membrane potential (*x* axis) is represented as the percentage from the highest membrane potential in state 4 of respiration. Results are means±S.E.M. of three independent experiments performed at least in duplicate.

As a measure of respiratory uncoupling, we assessed the proton conductance of spheroplasts from yeast overexpressing wild type and the mutated UCP2 proteins carrying the amino acid changes found in patient 1 and patient 2. Spheroplasts from yeast transformed with the empty vector were used as control. The results are depicted in Figure 3. As it has been described previously [12], [36], overexpression of wild type UCP2 in yeast mitochondria shifted the curve upward and to the left, which is consistent with an increase of the proton leak and thus uncoupling of mitochondrial respiration, compared to the control spheroplasts. Expression of the mutated forms of UCP2, patient 1 or patient 2, also uncoupled mitochondrial respiration when compared to control spheroplasts. However, yeasts expressing patient 1 and patient 2 UCP2 proteins, showed lower proton permeability compared to the ones expressing wild type UCP2, suggesting that both mutated proteins were less active than wild type UCP2. The same results were obtained with isolated mitochondria (data not shown).

### Functional studies in insulin-secreting cells

**Figure 4:**
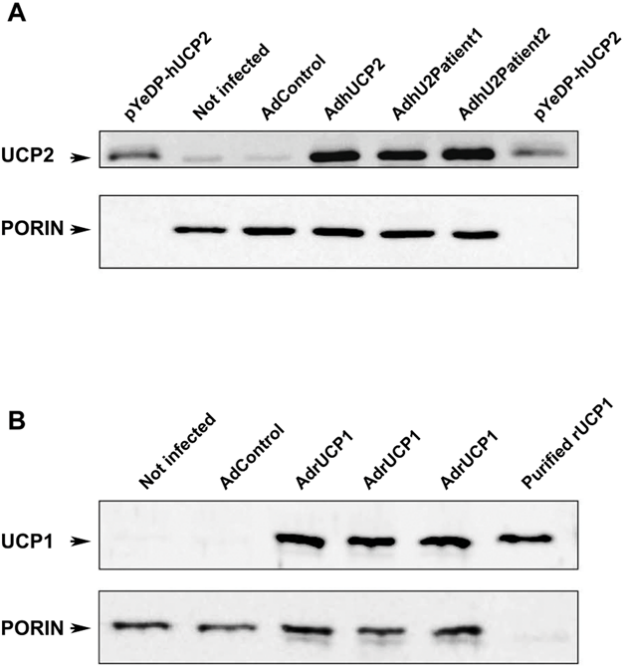
Expression levels of wild type, mutated UCP2 and UCP1 in mitochondrial extracts isolated from insulinoma INS-1E cells after adenoviral infection. Gels were loaded with 10 µg of mitochondrial proteins and analyzed by Western blot. A) INS-1E cells were infected with control adenovirus (Adcontrol, containing the GFP gene and the inverted sequence of human UCP2 cDNA at 5.2×10^6^ plaque-forming units (pfu)/ml) or with wild type (AdhUCP2 at 5.2×10^6^ pfu/ml) or mutated (AdhU2Patient1 at 1.3×10^6^ pfu/ml and AdhU2Patient2 at 2.6×10^6^ pfu/ml) UCP2 adenovirus. Mitochondria-rich fraction from not infected INS-1E cells was also used as control of endogenous UCP2 expression. 1 µg of mitochondrial proteins from yeasts overexpressing human UCP2 (pYeDP-hUCP2) were used as positive control. UCP2 protein was revealed using anti-hUCP2 antibody [31] and anti-UCP2 (C-20) (Santa Cruz Biotechnology, Inc, Santa Cruz, CA). As a control of similar amount of proteins loaded, the membrane was reprobed with anti-VDAC/Porin antibody (Sigma-Aldrich, St Louis, MO, USA). B) INS-1E cells were infected with control (Adcontrol, containing the GFP gene and the inverted sequence of human UCP2 cDNA) or with rat UCP1 adenovirus (AdrUCP1) both at a concentration of 5.2×10^6^ pfu/ml. Mitochondria-rich fraction from not infected INS-1E cells was also used as control of endogenous expression. 40 ng of purified rat UCP1 were used as positive control. UCP1 protein was revealed using a polyclonal anti-rat UCP1 antibody prepared in the laboratory. As a control of similar amount of proteins loaded, the membrane was reprobed with anti-VDAC/Porin antibody (Sigma-Aldrich, St Louis, MO, USA).

**Figure 5:**
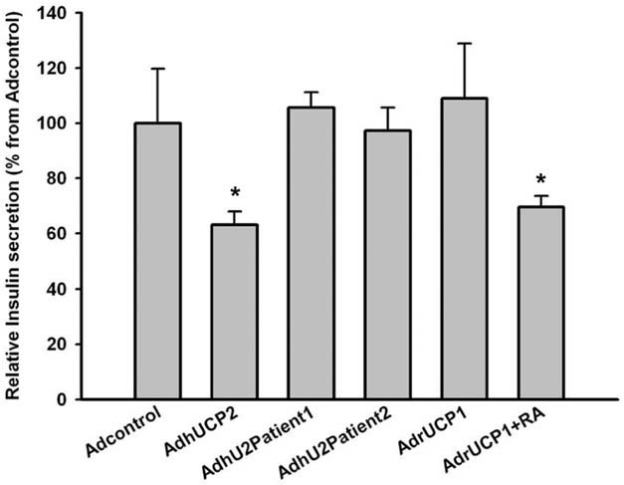
Effect of UCP2 mutations on insulin secretion of INS-1E cells in response to glucose. Insulin release by INS-1E cells expressing adenoviral wild type (AdhUCP2), mutated (AdhU2Patient1 and AdhU2Patient2) UCP2 or rat UCP1 (AdrUCP1) compared to control (Adcontrol, containing the GFP gene and the inverted sequence of human UCP2 cDNA). Values represent insulin secreted into the medium as percentage from the insulin secreted by cells infected with control adenovirus (Adcontrol) in the presence of 15 mM glucose (for details see “Materials and Methods” section). 32 µM retinoic acid (RA) was added to the cultures of AdUCP1 infected cells to activate UCP1 [36]. Data are means±S.E.M. of at least 6 independent experiments performed in duplicate. The level of statistical significance of the differences between cells infected with control adenovirus and with UCP2 or UCP1 adenovirus is indicated: ** *p*<0.001 (*t* test).

To assess whether the modification of UCP2 activity observed in patients 1 and 2 could affect insulin secretion in beta cells, we subjected an insulin-secreting pancreatic insulinoma cell line (INS-1E) [35] to adenoviral expression of wild type and mutated UCP2 in mitochondria. Adenovirus expressing rat UCP1 was used as control of uncoupling protein. The adenovirus concentration was adjusted for each construct in order to obtain similar amounts of the UCP2 proteins in the INS-1E cell mitochondria (assessed by western blot, Figure 4). At the concentrations employed for each adenoviral construct (see methods section), more than 95% of the cells were infected and expressed GFP, as evidenced by flow cytometry and fluorescence microscopy (data not shown). Infection of INS-1E cells by the recombinant adenoviruses, Ad-hUCP2, Ad-hU2Patient1and Ad-hU2Patient2, enhanced the mitochondrial content of the corresponding proteins around 15 times compared with that of control cells infected with the highest concentration of adenovirus containing the GFP and the inverted UCP2 cDNA (Figure 4). Insulin secretion by INS-1E cells expressing UCP1, wild type and mutant UCP2 proteins was determined in the presence of 15 mM glucose. Results are shown in Figure 5. As expected, adenoviral UCP2 infection significantly reduced insulin release by INS-1E cells (nearly 40% compared to infection with control adenovirus). In contrast, infection of INS-1E cells with AdhU2Patient 1 or AdhU2Patient 2 did not modify the insulin secretion levels from control values. Similar results were obtained with cells overexpressing UCP1. Actually, this was probably due to the high concentration of cytosolic nucleotides that keeps UCP1 activity inhibited since a decrease of near 40% of the insulin release was observed in INS-1E cells overexpressing UCP1, when UCP1 activity was stimulated by 32 µM retinoic acid (Figure 5), a known activator of UCP1 [36].

## Discussion

Although most severe CHI are due to mutations in both genes *ABCC8* and *KCNJ11* [1]–[3], other mutations have also been identified in the glucokinase gene (*GCK*) [4], the glutamate dehydrogenase gene [5], (*GLUD1*), the short-chain L-3-hydroxyacyl-CoA dehydrogenase gene (SCHAD) [6], the insulin receptor gene [7], the *HNF4A* gene in the setting of maturity-onset diabetes (MODY) [8], and the *SLC16A1* gene which encodes monocarboxylate transporter 1 (MCT1) in the setting of exercise-induced hypoglycaemia [9]. Nevertheless, a large number of CHI cases remain unexplained. The importance of ATP level or ATP/ADP ratio in the control of insulin secretion indicates that mitochondria and oxidative metabolism may play an important role in this event. Interestingly, *UCP2*, which encodes a mitochondrial transporter, was initially presented as a gene possibly linked to obesity and hyperinsulinism in mice since it maps to regions of human chromosome 11 and mouse chromosome 7 that have been linked to hyperinsulinemia and obesity [12].

In agreement with an expected role for UCP2 in the regulation of insulin secretion, its ability to inhibit insulin secretion in animals, although it was questionned [37], was largely documented both using experimental modulations of UCP2 level [15]–[18], [21] or activity [22] in insulin-secreting cells and analysis of *Ucp2*-null mice [23]. In addition, other animal studies suggested that UCP2-mediated control of insulin secretion is a physiologically-relevant mechanism of the metabolic response to fasting since UCP2 has been proposed to keep insulin secretion low during starvation [38], [39], this function being under the control of the transcription co-repressor, surtuin-1 [40], [41]. UCP2 negatively regulates glucose sensing in hypothalamic neurons and participates to metabolic switch from glucose metabolism to fatty acid oxidation during starvation [42], [43]. In addition to a possible role as a regulator of ATP level, it has also been proposed that UCP2 could either act through a modulation of ROS levels by mitochondrial activity [15], [44], [45], or through a regulation of mitochondrial Ca^2+^ uptake [46]. Undoubtedly, there is still an uncertainty regarding the mechanism explaining the activity of UCP2 and other novel UCPs [47] and in the present study we did not investigate the precise mechanism allowing UCP2 to inhibit insulin secretion. This question will require further studies.

Despite many animal studies, no report of a role for *UCP2* in insulin secretion in humans was described. The data obtained in mice prompted us to look for UCP2 mutations in patients presenting abnormal insulin secretion. The familial pattern of moderate hyperinsulinism noted in the present study, with more severe phenotype in children than in the carrier parent, suggested an autosomal dominant inheritance with variable expressivity and/or incomplete penetrance. However, it is important to keep in mind that within moderate forms of hyperinsulinism, hypoglycemia can be undiagnosed. Similar intrafamiliar variability was also reported in dominant forms of hyperinsulinism related to other genes such as *SUR1* [48], [49], *GCK* [4] and *GLUD1* [5]. As far as *UCP2* is concerned, age-depending genetic and environmental factors may be involved in its regulation. According with this, it has been shown in mice, that *Ucp2* expression increased after birth and decreased later on in the life [50]. In addition, as newborns and infants have a higher nutritional intake (in g/kg body weight and day) than adults, and as glucose and fatty acids increased the *Ucp2* transcription [19], UCP2 dysfunction may have higher physiopathological implications in children than in adults.

The two procedures used to assay the activity of the *UCP2* mutants, expression in yeast or expression in insulinoma cells, were previously used by us and others [12], [16], [18], [36]. Taken together, these data show that the mutations identified from 2 CHI patients are associated with a loss-of-function of the UCP2 protein corresponding to a loss of uncoupling activity seen in yeast. As a result, whereas the wild type UCP2 inhibits insulin secretion by pancreatic beta cells, the mutated UCP2 promote insulin secretion and as such can explain the hyperinsulinism observed in these patients.

This is the first time that a role for *UCP2* in insulin secretion is shown to be relevant in human disease. These novel findings may contribute to define the pathogenesis and genetic origins of congenital hyperinsulinism which remains largely unexplained in more than half of all patients, and could have wider implications for unexplained hypoglycaemia and impaired glucose sensitivity. These data demonstrate that UCP2 should be added to the growing list of genes in which mutations can lead to CHI, and introduce UCP2 as a new player in the control of insulin secretion and glucose sensing by ß-cells in humans. In addition, these results also reinforce the critical role played by mitochondria in the control of insulin secretion.
